# Light dominates the diurnal emissions of herbivore-induced volatiles in wild tobacco

**DOI:** 10.1186/s12870-021-03179-z

**Published:** 2021-08-30

**Authors:** Jun He, Rayko Halitschke, Meredith C. Schuman, Ian T. Baldwin

**Affiliations:** 1 National Citrus Engineering Research Center, Citrus Research Institute, Southwest University, Xiema Street, Beibei, Chongqing, 400712 People’s Republic of China; 2 Department of Molecular Ecology, Max Planck Institute for Chemical Ecology, Hans-Knöll-Straße 8, 07745 Jena, Germany; 3 Current address: Departments of Geography and Chemistry, University of Zurich, 8057 Zürich, Switzerland

**Keywords:** Plant diurnal rhythms, Herbivory, Light regulation, Terpenoid volatiles, GLVs, HIPVs

## Abstract

**Background:**

Timing is everything when it comes to the fitness outcome of a plant’s ecological interactions, and accurate timing is particularly relevant for interactions with herbivores or mutualists that are based on ephemeral emissions of volatile organic compounds. Previous studies of the wild tobacco *N. attenuata* have found associations between the diurnal timing of volatile emissions, and daytime predation of herbivores by their natural enemies.

**Results:**

Here, we investigated the role of light in regulating two biosynthetic groups of volatiles, terpenoids and green leaf volatiles (GLVs), which dominate the herbivore-induced bouquet of *N. attenuata*. Light deprivation strongly suppressed terpenoid emissions while enhancing GLV emissions, albeit with a time lag. Silencing the expression of photoreceptor genes did not alter terpenoid emission rhythms, but silencing expression of the phytochrome gene, *NaPhyB1*, disordered the emission of the GLV (*Z*)-3-hexenyl acetate. External abscisic acid (ABA) treatments increased stomatal resistance, but did not truncate the emission of terpenoid volatiles (recovered in the headspace). However, ABA treatment enhanced GLV emissions and leaf internal pools (recovered from tissue), and reduced internal linalool pools. In contrast to the pattern of diurnal terpenoid emissions and nocturnal GLV emissions, transcripts of herbivore-induced plant volatile (HIPV) biosynthetic genes peaked during the day. The promotor regions of these genes were populated with various *cis*-acting regulatory elements involved in light-, stress-, phytohormone- and circadian regulation.

**Conclusions:**

This research provides insights into the complexity of the mechanisms involved in the regulation of HIPV bouquets, a mechanistic complexity which rivals the functional complexity of HIPVs, which includes repelling herbivores, calling for body guards, and attracting pollinators.

**Supplementary Information:**

The online version contains supplementary material available at 10.1186/s12870-021-03179-z.

## Background

Living on a planet that rotates on its axis every 24 h, most organisms’ physiologies harbor rhythms that are tuned to periodic day/night cycles. For example, the plant circadian clock prepares the photosynthetic machinery for light harvesting just before sunrise and closes stomata to maintain water at noon [1]. Plant rhythms are also important in coordinating interactions with other organisms. For instance, snapdragon flowers diurnally emit methyl benzoate to attract day-active pollinating bees [2] but petunia flowers nocturnally emit benzaldehyde to attract night-active hawkmoths [3]. Diurnal rhythms in jasmonate signaling of *A. thaliana* are thought to be essential for its resistance against generalist cabbage loopers, *Trichoplusia ni,* which also feed rhythmically [4]. Both resistance of *A. thaliana* and infection by *Botrytis cinerea* vary by time of day [5, 6].

Herbivore-induced plant volatiles (HIPVs) mediate tri-trophic interactions between plants, herbivores and natural enemies [7–9]. Plants use HIPVs to attract predators of herbivores and to indirectly defend themselves from herbivory. However, herbivores can also use these volatiles to locate their host plants or avoid predation [7, 8]. HIPVs usually comprise two ubiquitous groups of metabolites: green leaf volatiles (GLVs) and terpenoids. GLVs are C_6_ aldehydes, alcohols and esters, which are synthesized through the lipoxygenase (LOX)/hydroperoxide lyase (HPL) pathway in almost all plants and algae [10–14]. Terpenoids are synthesized from different numbers of C_5_ units and are classified into hemiterpene (C_5_), monoterpene (C_10_), sesquiterpene (C_15_) and diterpene (C_20_), backbones and their derivatives [11, 15]. Terpenoid volatiles are derived from mevalonate (MVA) or the 2-C-methyl-D-erythritol 4-phosphate/ 1-deoxy-D-xylulose 5-phosphate (MEP/DOXP) pathway in higher plants [16], and directly synthesized by terpene synthases [15]. Both GLVs and terpenoids play roles in tri-trophic interactions, some of which function as indirect defenses for plants [7, 8].

HIPVs released from foliage are known to be diurnally regulated, particularly the terpenoid constituents. For example, the abundance of volatiles emitted from *Picea abies* foliage after methyl jasmonate elicitation, which mimic herbivore-induced emissions, is greater during day than night [17]. Similar patterns are reported for *Phaseolus vulgaris* HIPVs elicited by attack from *L. huidobrensis* larvae [18]. Furthermore, in *A. annua* the gene *QH6*, which encodes (−)-α-PINENE/(−)-β-PINENE SYNTHASE, showed diurnal patterns of transcript abundance [19]. GLV emissions were induced by herbivory both during the day and during the night from *Phaseolus lunatus* [20], during night from *Nicotiana tabacum* [21], while in *P. vulgaris* (kidney bean), some constituents are released during light periods while others are released during dark periods [18].

In previous research with the native diploid tobacco *N. attenuata*, we found that foliar HIPV constituents, including (*E*)-α-bergamotene, (*E*)-β-ocimene and linalool, were diurnally released [22–25]. These volatiles play a central role in *N. attenuata’s* herbivory, defense and pollination interactions with insects and the same compounds play different roles depending on the tissue from which they are released and their timing. For example, (*E*)-α-bergamotene, produced by *NaTPS38*, is emitted from flowers at night and increases the probing time and thus pollination success of *M. sexta* moths visiting flowers. However, when emitted from attacked leaves during the day, it attracts natural enemies of *M. sexta* larvae [24] to mediate a potent indirect defense [26]. The emission of (*E*)-β-ocimene, produced by *NaTPS25*, was correlated with (*E*)-α-bergamotene emission from diverse *N. attenuata* accessions, and the two genes are co-localized on the *N. attenuata* genome [27]. In contrast, a QTL for the emission of linalool, a monoterpene produced by *NaLIS*, was mapped to a locus different from that of (*E*)-α-bergamotene and (*E*)-β-ocimene. Linalool also attracts natural enemies, and can deter oviposition of *M. sexta* moths in a context-dependent manner [23]. GLVs have also been found to play important roles in the defense of *N. attenuata* against herbivory in nature [26]*.* GLVs released in the morning elicit greater predatory activity by day-active *Geocoris* spp. predators than does the evening-released blend of GLVs even when both blends are experimentally applied to plants during the window of *Geocoris* spp. activity [28]. Furthermore, transcripts of genes involved in GLV biosynthesis, including *NaHPL* and *NaLOX2,* were regulated by the plant’s circadian clock [25, 29].

Here we confirmed that terpenoid volatile emission is greater during light periods while emission of GLVs is greater during dark periods, by manipulating the light environment of leaves independently of day-night cycles. The emission patterns of terpenoids were not altered by silencing the expression of photoreceptor genes, the supplementation of far-red light, or by increasing stomatal resistance by external abscisic acid (ABA) application. The emission pattern of GLVs, however, was changed by silencing *NaPhyB1* transcripts, and increased by exogenous ABA treatments. The genes involved in terpenoid and GLV biosynthesis all showed diurnal patterns of transcript accumulation, and the promoter regions of these genes were populated by various *cis*-acting regulatory elements involved in light-, stress-, phytohormone- or circadian-regulated processes.

## Results

### Light deprivation decreased terpenoid, but increased GLV emissions

**Fig. 1:**
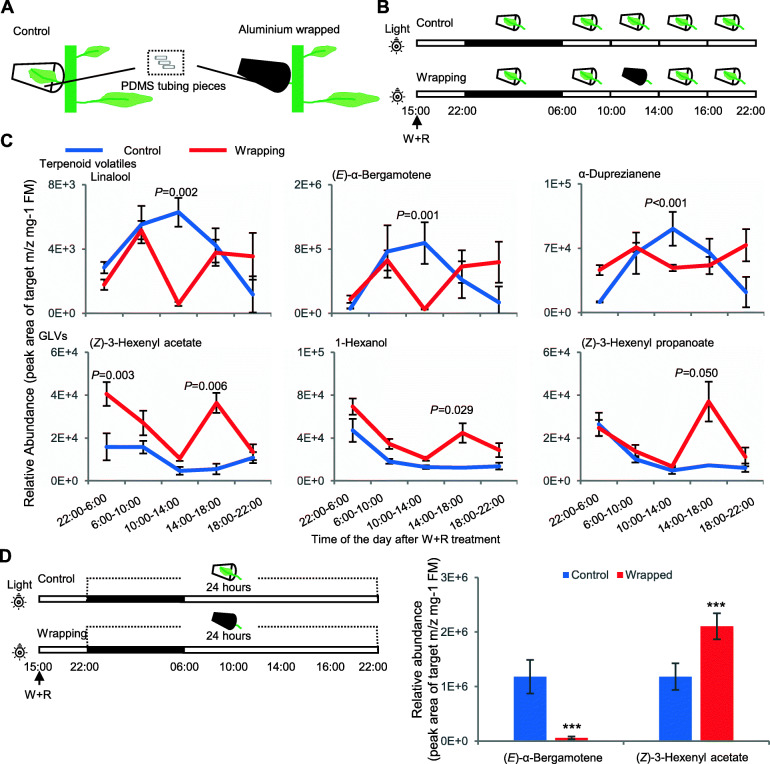
Light deprivation suppressed the emission of terpenoid volatiles but increased GLV emissions after W + R treatments. (**A**) Collection of headspace volatiles with PDMS tubing pieces from *N. attenuata* leaves enclosed in plastic cups [30]. The target leaf was deprived of light by wrapping the outside of the cup with aluminum foil. (**B**) Experimental set-up for volatile collections after W + R treatment during night, day and temporary light deprivation. Each block represents an independent collection. Black bars indicate the night and white bars indicate day. Wrapped cups (black) provided the local light deprivation. (**C**) Dynamic induction of representative volatiles by W + R treatment and alterations by temporary local light deprivation. (**D**) Setup for 24 h light deprivation and volatile collection (right), and suppression of terpenoid emission and enhancement of GLV emission (left), Student *t*-test, *n* = 3, ***: *p* < 0.001

Predation assays performed with a biparential RIL population of *N. attenuata* plants grown in a field plot in the summer of 2017 revealed that *M. sexta* eggs and larvae were predated by big-eyed bugs (*Geocoris* spp.) during the day time (Fig. S1), consistent with previous findings [27]. Because plant volatiles play essential roles in mediating this tri-trophic interaction [23, 26] and to get a better understanding of their diurnal/nocturnal regulation, we collected headspace day and night time volatiles from *N. attenuata* leaves on glasshouse-grown plants elicited by wounding plus *M. sexta* larval regurgitant (W + R) using PDMS tubing pieces (Kallenbach et al. [30], Fig. 1A, B). The assayed leaf was enclosed in a plastic cup which could be wrapped using aluminum foil to locally deprive the treated leaf of light (Fig. 1A, B). We found that the emission of most terpenoid volatiles corresponded strongly to light periods, especially linalool and (*E*)-α-bergamotene (Fig. 1C). The emission of these terpenoid volatiles was very low during the night period (22:00–6:00), increased in the morning (6:00–10:00), and peaked during the middle of the day (10:00–14:00), then decreased in the afternoon (14:00–18:00) and returned to low levels in the evening (18:00–22:00). When the leaf was enclosed in a wrapped cup during the middle hours of the day (10:00–14:00), the emission of most terpenoids immediately decreased to low levels, comparable to those measured at night. After the foil wrapping was removed in the afternoon and evening, the emission of these terpenoid volatiles returned to control levels (Fig. 1C). In contrast to the day-emitted terpenoids, many GLVs were abundantly emitted at night and steadily decreased during the day, although some, such as (*Z*)-3-hexenyl acetate and (*Z*)-3-hexenyl propanoate, continued to be released at high levels in the morning. Local light deprivation during the middle hours of the day did not alter GLV emissions but up-regulated them in the afternoon, which likely continued through the evening hours (Fig. 1C). The decreased total terpenoid volatile emissions and increased total GLV emissions associated with light deprivation were further confirmed in single 24 h collections after W + R treatments (Fig. 1D). Thus terpenoids and GLVs showed opposing responses to local light deprivation.

Because the volatile emission might also be affected by temperature and humidity caused by wrapping the cups, we monitored these two factors outside of cups, in clean cups and in wrapped cups (Fig. S2A). We found that there was only 0.8–2.4 °C drop in temperature in wrapped cups compared to clean cups during the wrapping period 10:00–14:00 (Fig. S2B). The average humidity in wrapped cups were about 9% higher than in clean cups, but it was more variable depending on the enclosed leaf (Fig. S2B). These difference in temperature and humidity were not supposed to significantly affect the volatile emission in our experimental setups because they were in the comfortable zone of the plants.

### Silencing *NaPhyB1* expression and ABA treatments altered GLV but not terpenoid emissions

**Fig. 2:**
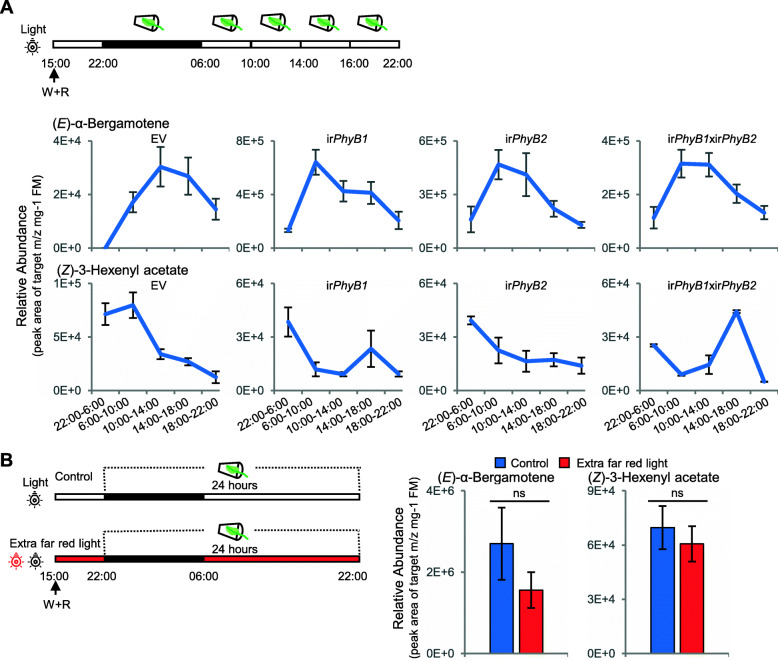
Silencing photoreceptor genes changed the rhythm of GLV (*Z*)-3-hexenyl acetate but not terpenoid (*E*)-α-bergamotene. (**A**) Dynamic volatile collections and measurements of (*E*)-α-bergamotene and (*Z*)-3-hexenyl acetate in *N. attenuata* lines with different photoreceptor gene silenced by RNAi (inverted repeat:ir). (**B**) Supplementation with extra far-red light did not alter the abundance of emitted volatiles during 24 h collections

We assessed the volatile emissions elicited by W + R treatment in a series of plants individually silenced (by stable transformation with inverted repeat—ir—RNAi constructs) in the following photoreceptor genes: *NaPhyB1, NaPhyB2, NaPhyB1 x NaPhyB2, NaCrypt1 and NaCrypt2* (previously characterized by Oh et al. [31]). The emission pattern of volatiles in all the plants were largely unchanged (Fig. 2A, Fig. S3) with the exception of those of ir*PhyB1* plants. Particularly, in plants generated by crossing ir*PhyB1* with ir*PhyB2* to silence both phytochrome genes, (*Z*)-3-hexenyl acetate emissions increased in the afternoon. Supplementation of far red light did not alter total terpenoid or GLV emissions as assessed in 24 h collections (Fig. 2B) or collections taken every 4 h (Fig. S4).

### ABA regulated HIPVs but not via stomatal conductance

**Fig. 3:**
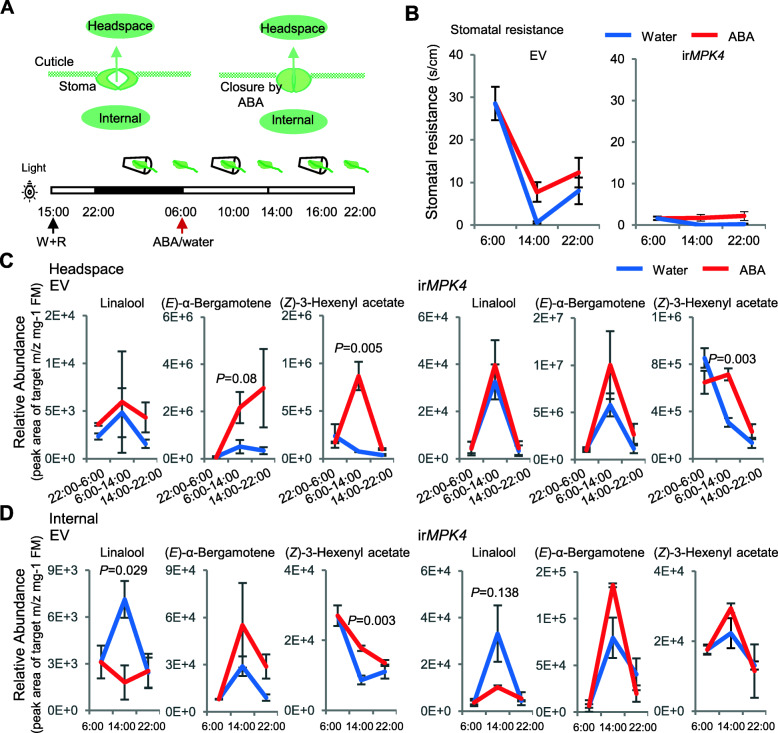
ABA treatment altered stomatal resistance, and emission and internal storage of GLV and terpenoid volatiles. (**A**) The proposed set-up to test for evidence of emission of volatiles through stoma (upper panel), and setup for ABA treatment and volatile collection (lower panel). Time span for collection of headspace volatiles is indicated by a leaf in a cup and time point for internal volatiles is indicated by a leaf without a cup. (**B**) Dynamic stomatal resistance in the leaf of EV and ir*MPK4* plants. (**C, D**) linalool, (*E*)-α-bergamotene and (*Z*)-3-hexenyl acetate in headspace (**C**) and in internal leaf pools (**D**) in the leaf of EV and ir*MPK4* plants, in which stomata tend not to close. Time points with significant (or close to significant) difference are indicated with *p-*values from Student *t*-tests, *n* = 3

We tested if the day/night patterns of volatile emission were controlled by changes in stomatal resistance, which might influence the release of volatiles from plant tissues (Fig. 3A, [32, 33]). We used ABA treatments to increase the stomatal resistance of leaves of empty vector control (EV) and ir*MPK4* plants, which, as previously described [34], have stomata that are largely in the open state and are not responsive to ABA treatments (Fig. 3A, B). ABA treatment of EV plants increased stomatal resistance during the day, but the diurnal emissions of the terpenoids, linalool and (*E*)-α-bergamotene, were not altered in either EV or ir*MPK4* plants (Fig. 3C). In contrast, emissions of (*Z*)-3-hexenyl acetate were upregulated at the sampling period that followed the ABA treatment and returned to control levels at the end of the day in both EV and ir*MPK4* plants (Fig. 3C). Interestingly, measurements of the internal leaf volatile pools revealed that ABA treatments decreased the amounts of linalool accumulated in leaves during the middle part of the day in both EV and ir*MPK4* plants, although it did not decrease amounts released to the headspace (Fig. 3C, D). Internal (*E*)-α-bergamotene pools tended to increase but were not significantly changed by ABA treatment, consistent with the headspace emissions. Internal (*Z*)-3-hexenyl acetate pools tracked headspace emissions, and both were enhanced by ABA treatments in EV plants, with smaller enhancements in ir*MPK4* plants (Fig. 3D).

Thus ABA treatment enhanced the emission of a GLV, which is more abundantly emitted in the dark, despite reducing stomatal conductance. Similarly, impaired red/far red light perception also increased GLV emission. However, neither ABA treatment nor impairment of plant light perception had a significant effect on emission of terpenoids.

### Genes responsible for terpenoid and GLV biosynthesis are diurnally transcribed

**Fig. 4:**
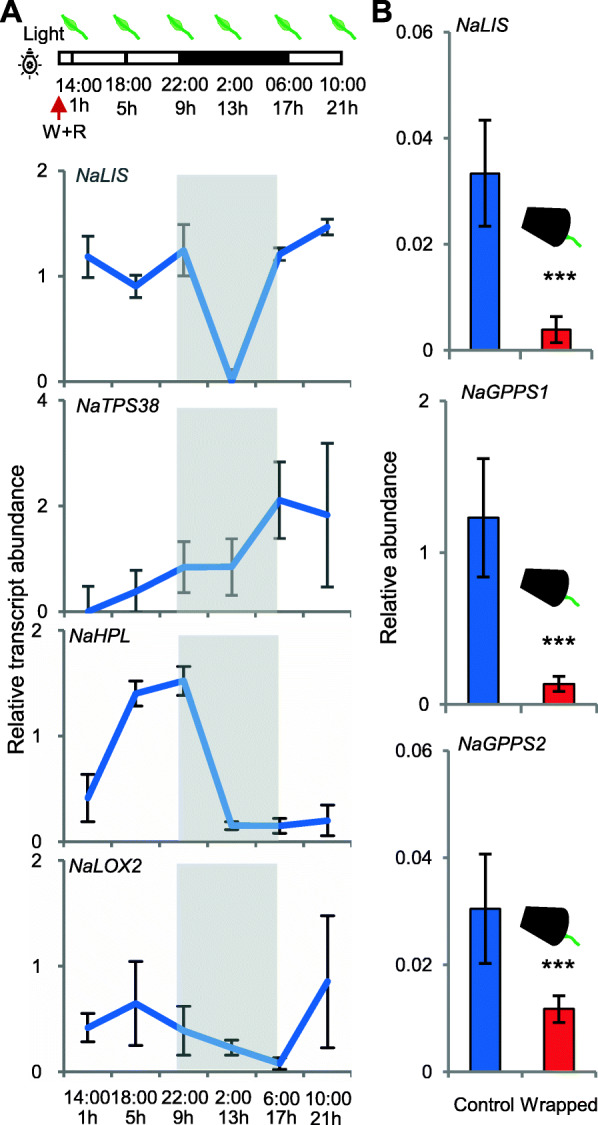
Transcription of genes involved in biosynthesis of terpenoids and GLVs corresponding to illumination. (**A**) Transcript abundance of linalool synthase gene *NaLIS* and (*E*)-α-bergamotene synthase gene *NaTPS38,* and *NaHPL* and *NaLOX2*, which are involved in the biosynthesis of GLVs during 21 h after W + R treatments. Data are from a published microarray data set (Kim et al. 2011). (**B**) Transcript abundance of *NaLIS* and two *GPPSs*, which are responsible for biosynthesis of the monoterpenoid substrate GPP, are down-regulated in leaves enclosed in foil-wrapped cups. Measured with qRT-PCR

A previously published microarray data set revealed dynamic transcriptome changes in *N. attenuata* after W + R treatment [35]. From this data set, *NaLIS* transcripts, encoding the linalool synthase 23, were abundant in the daytime and barely detectable at night (Fig. 4A). Transcripts of the (*E*)-α-bergamotene synthase gene, *NaTPS38* [24]*,* were strongly increased in both day and night, however, the rate of up-regulation was greater during the day. Intriguingly, transcripts of *NaHPL* and *NaLOX2,* both involved in GLV biosynthesis, were also more abundant during the day, even though the emission of most GLVs is nocturnal. Using qRT-PCR we further confirmed that light deprivation strongly down-regulated *NaLIS* transcript levels, as well as those of *NaGPPS1* and *NaGPPS2,* genes responsible for the biosynthesis of monoterpenoid substrates upstream in the pathway (Fig. 4B).

### *Cis*-acting regulatory elements involved in light-, stress-, phytohormone- and circadian-regulation were identified in the biosynthetic genes

**Fig. 5:**
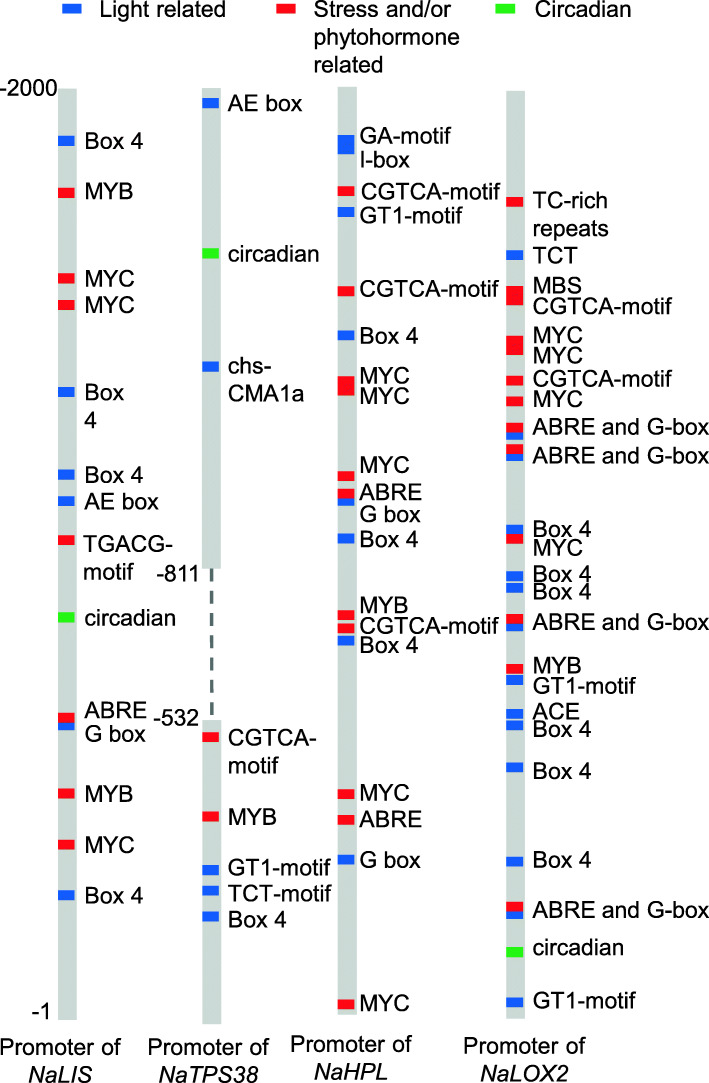
*Cis*-acting regulatory elements in promoter regions of the four biosynthetic genes. The 2Kb regions upstream start codons of *NaLIS*, *NaTPS38*, *NaHPL* and *NaLOX2* extracted from the genomic sequences of *N. attenuata* were searched in the Plant *Cis*-acting Regulatory Element online database (http://bioinformatics.psb.ugent.be/webtools/plantcare/html/, Lescot et al. [44])

We scanned the 2 kb promoter regions of *NaLIS, NaTPS38, NaHPL* and *NaLOX2* for *cis*-acting regulatory elements (Fig. 5, Table 1, Table S2). *NaLIS* has six and *NaTPS38* five elements identified as potentially involved in responsiveness to light in their promoters, and eight and fourteen were found in the promotors of *NaHPL* and *NaLOX2*, respectively. Box 4 [36] was present in all the promoters with different copy numbers. Some of the elements were found in two or three of the promoters such as the AE box [37], G-box [38, 39], GT1-motif [40], and TCT-motif [41]. The others were only found in a specific promoter, e.g. the ACE [42] element was only in the promoter of *NaLOX*, chs-CMA1a [43] in the promoter of *NaLIS*, GA-motif [44] and I-box [45] in the promoter of *NaHPL*. The promoter regions of these genes also contained numerous elements responsive tostress and/or phytohormones including JA, MeJA, SA and ABA, specifically: seven for *NaLIS*, eight for *NaTPS38*, 11 for *NaHPL* and 13 for *NaLOX*. Notably, the ABRE element (ACGTG/CACGTG/AACCCGG) involved in ABA regulation [46, 47] was specifically found in the *NaLIS, NaHPL* and *NaLOX2* promotor regions, but not for *NaTPS38*. This is consistent with ABA treatment enhancing (*Z*)-3-hexenyl acetate emissions and internal pools, and decreasing linalool internal pools, but not affecting (*E*)-α-bergamotene (Fig. 3D). A single circadian element (CAAAGATATC, [48]) was identified for *NaLIS*, *NaTPS38*, and *NaLOX2,* but not for *NaHPL*.

**Table 1:** *Cis*-acting regulatory elements identified in the promoter sequences of *NaLIS*, *NaTPS38*, *NaHPL* and *NaLOX*

Name	Consensus sequences	***Number of copies***				Stimuli	Reference
		***Na*** LIS*	***Na*** TPS38*	***Na*** ***HPL***	***Na*** ***LOX***		
ACE	GACACGTATG				1	light	[42]
AE-box	AGAAACAA	1	1			light	[37]
Box 4	ATTAAT	4	1	3	6	light	[36]
chs-CMA1a	TTACTTAA		1			light	[43]
GA-motif	ATAGATAA			1		light	[44]
G-box	CACGTC/CACGTG/	1		2	4	light	[38, 39]
	CACGAC/						
	CACGTT/TAAACGTG						
GT1-motif	GGTTAA		1	1	2	light	[40]
I-box	AGATAAGG			1		light	[45]
TCT-motif	TCTTAC		1		1	light	[41]
MYB	CAACTG/CAACAG/	2	2	1	2	JA/stress	[49, 50]
	TAACTG/TAACCA						
MYC	CATGTG/TCTCTTA/	3	3	5	4	JA/stress	[49, 51]
	CAATTG/CATTTG						
CGTCA-motif	CGTCA		1	3	2	MeJA	[52, 53]
TGACG-motif	TGACG	1	1			MeJA	[52, 53]
ABRE	ACGTG/CACGTG/	1		2	4	ABA	[46, 47]
	AACCCGG						
TC-rich repeats	GTTTTCTTAC				1	stress	[54]
TCA-element	TCAGAAGAGG		1			SA	[55, 56]
circadian	CAAAGATATC	1	1		1	circadian	[48]

## Discussion

HIPVs emitted from *N. attenuata* plants varied in composition and abundance across the day/night cycle. Terpenoid constituents of the volatile bouquet showed emissions which peaked in the middle of the day and responded quickly to light deprivation, while GLVs tended to be emitted more at night and were enhanced by light deprivation. Rhythms of the terpenoid volatiles were not altered by the constitutive silencing of photoreceptor genes, supplementation by far-red light, or stomatal closure elicited by ABA treatments. GLV emissions, including (*Z*)-3-hexenyl acetate, were increased by *PhyB* silencing and ABA treatments. Transcript abundances of the genes involved in the biosynthesis of terpenoids and GLVs varied over a day/night cycle, and various light- or JA-responsive *cis*-acting regulatory elements, as well as some circadian clock and ABA regulation elements were identified in their promoter regions.

### Correlations of HIPV emissions with herbivory and tri-trophic interactions

In nature, plants must cope with environments characterized by periodic changes that accompany day/night cycles that include fluctuations in many factors in addition to light: temperature, humidity, and innumerable biotic interactions. For instance, plants need to adopt to rhythms of attacks by pathogens and herbivores, or activity patterns of beneficial species, such as pollinators and natural enemies of herbivores [4, 6, 57, 58]. To cope with this environmental rhythmicity, plants have evolved both endogenous rhythms and the ability to coordinate responses to changes in specific environmental factors. Much of this can be seen in changes in the abundance and composition of their metabolites [59]. Different HIPV constituents of *N. attenuata* plants are associated with activities of different species that interact with the plants in nature. The larvae of the specialist *Manduca* spp. are known to feed diurnally. Experimental measures of predation rates of eggs and larvae were correlated with the diurnal emissions of terpenoid volatiles, which is consistent with previous reports by Joo et al. [28]. If we understand the mechanisms responsible for these emission patterns, these mechanisms could provide means of experimentally manipulating the patterns and hence provide a way to test such correlations observed in field studies.

### Rhythms of the emission of terpenoid volatiles and GLVs

Herbivore-induced mono- or sesquiterpene terpenoids are diurnally regulated in many species such as *G. hirsutum* [60], *A. annua* [19], *A. majus* [61] and *Phaseolus vulgaris* [18] upon herbivory. Similarly, in *N. attenuata* at least (*E*)-β-ocimene, (*E*)-α-bergamotene and linalool showed diurnal emission following herbivore elicitation [22, 23]. Interestingly, (*E*)-α-bergamotene is diurnally emitted from foliage of *N. attenuata* plants and functions in attracting natural enemies that protect plants from herbivory by larvae of *M. sexta*. However, the same compound is nocturnally released from flowers and attracts adults of the same insect, which provides pollination services [24]. Further study showed that (*E*)-β-ocimene and (*E*)-α-bergamotene are correlated with each other across different *N. attenuata* genotypes, but linalool is independently regulated [23, 27]. Furthermore, unlike the emission of linalool, the endogenous leaf pools of conjugates derived from linalool, such as linalool glycosides, lack the diurnal pattern of emissions [23].

In this study we confirmed the diurnal emission pattern of terpenoid volatiles, including (*E*)-α-bergamotene and linalool [28], and further found that this diurnal pattern was not altered by the silencing of photoreceptor genes or increased stomatal resistance resulting from ABA treatments. Interestingly, ABA treatment suppressed internal linalool, but had little influence on internal (*E*)-α-bergamotene. The contradictory influences of ABA treatments on emission and internal linalool might be attributed to opposing effects of ABA on biosynthesis or conjugation of linalool, and the mechanisms of the emission process.

In contrast to conserved diurnal emission of terpenoids across different species, GLVs showed both diurnal and nocturnal release. For example, mechanical damage to lima bean (*Phaseolus lunatus*) plants during the light phase induced high emissions of both β-ocimene and (*Z*)-3-hexenyl acetate, while damage during the night only upregulated the emission of (*Z*)-3-hexenyl acetate [20]. In elicited kidney bean, GLV (*Z*)-3-hexenol was abundantly emitted during the night while the opposite was true for (*Z*)-3-hexenyl acetate [18]. In this study, we revealed a high nocturnal emission of GLVs, which is in agreement with previous studies [28, 29]. Additional work is required to understand the potential ecological significance of these opposing patterns for different constituents, and for these inferences, basic natural history information about the *N. attenuata* plants and activities of their surrounding organisms in the field plays a central role. For example, GLVs emitted during night might be used as feeding cues by nocturnally active herbivores in the field, as has been shown for *M. sexta* larvae in both laboratory and field studies [62].

### Light is essential for the regulation of HIPVs

A major conclusion of this study is that light strongly and locally regulates HIPV emissions, for both terpenoids and GLVs, although this could not be attributed directly to specific photoreceptors. This finding is consistent with earlier reports demonstrating that HIPV emissions from maize plants were light-dependent [63]. Light could influence the biosynthesis of terpenoids in at least two ways. First, pyruvate and glyceraldehyde 3-phosphate, the precursors for terpene biosynthesis, are derived from the Calvin cycle [64, 65], which requires the immediate products of photosynthesis. For example, biosynthesis of β-ocimene in lima bean plants dependeds on the CO_2_ fixation by photosynthesis [20]. Perhaps this explains the immediate reduction of terpenoid emission by light deprivation in our study, because it shuts down the primary source of substrates for the terpenoid biosynthesis pathway. However, in the sink parts of a plant, such as floral tissues, the biosynthesis of terpenoids is not limited by photosynthesis, and *N. attenuata* flowers emit (*E*)-α-bergamotene during the night [24]. Second, light as signal, can regulate the expression of the genes involved in the biosynthesis of terpenoids. Light is necessary for the expression of genes in the MEP/DOXP-pathway, which synthesizes the terpenoid precursors [66]. In seedlings of *A. thaliana* almost all the genes in the pathway are repressed in darkness [67]. In the current study, we also found that synthase genes of (*E*)-α-bergamotene, linalool, and the precursor GPP had lower transcript levels or were less induced by herbivory during night, were also downregulated by light deprivation in the middle of the day, and were associated with light-responsive regulatory elements.

Surprisingly, we found that GLVs were also strongly and temporarily affected by light and ABA treatment in *N. attenuata*. In response to herbivory, the increase in GLV emissions is more rapid than the response in terpenoid volatiles [28, 68]. Here, study, light deprivation in the middle of the day enhanced GLV emission with a delay during the subsequent hours, in contrast with the immediate reduction of terpenoid emission. This might be because the precursors for GLV biosynthesis, polyunsaturated fatty acids, are not immediate de novo products of photosynthesis. Therefore, light, probably together with the circadian clock, may affect biosynthesis of GLVs through regulating the expression of biosynthesis genes, with some delay in the accumulation of active proteins. This enhancement could be viewed as consistent with the effect of *NaPhyB1* silencing in the down-regulation of GLV (Z)-3-hexenyl acetate emissions, and suggests that far-red light could function as a signal regulating GLV emissions. However, volatile collections from leaves exposed to supplemental far-red light did not support this inference, implying that the regulation might be very transient or occur only at a specific interval of the day/night cycle. Furthermore, we found that transcript levels of *NaHPL* and *NaLOX2* sharply decreased at the start of the dark phase, in conflict with the higher nocturnal emission of most of the GLVs. These often contradictory results from physiological manipulations underscore the inference that emissions are under complex regulation. A detailed examination of the promotors of HIPV biosynthetic genes can be a valuable exercise so as to better understand the environmental factors that contribute to the patterns of transcript abundances.

### Complex regulation of HIPVs in *N. attenuata*

HIPV emissions are known to be influenced by herbivory elicitation [7], light conditions, the endogenous circadian clock [61], temperature [69], water availability [70] and the still mysterious emission process [71]. Many volatiles are only emitted or increased emission upon elicitation such as by herbivory and mechanical damage [67, 72, 73]. These inductions are usually mediated by the JA signaling pathway [11, 29, 74–78]. The circadian clock is another major regulator of HIPVs. For instance, genes involved in the biosynthesis of terpenoid volatiles, including *TPS*s and the genes upstream in the pathways, are known to be regulated by circadian clock components [61, 64, 65]. The transcription of GLV biosynthesis genes, *NaHPL* and *NaLOX2,* is also regulated by the circadian clock [25, 29].

The *cis*-acting regulatory elements identified in the promoter regions of the synthase genes of terpenoids and GLVs provide additional evidence for the concerted regulation of these genes by herbivory, light and the circadian clock. Multiple stress- and phytohormone-signaling elements were identified in the promoter regions of the genes involved in terpenoid and GLV biosynthesis and could provide a means of coordinating HIPVs with other JA-, MeJA, SA, and ABA-mediated chemical defenses. A number of light responsive elements and circadian related elements were found for both terpenoid and GLV genes. Interestingly, an ABA-responsive element present in the promoter of *NaLIS* but not *NaTPS38* might account for the specific suppression of *NaLIS* transcription and linalool biosynthesis by ABA treatments. These *cis*-acting regulatory elements suggests the regulation by different transcription factors that could coordinate HIPV biosynthesis with various environmental factors such as herbivory, light and the internal clock.

## Conclusions

In summary, this study revealed that light is necessary for the full induction of terpenoid volatiles and the suppression of GLVs, and regulates the transcription of HIPV genes in concert with many other factors.

## Materials and methods

### Plant materials and growth

Plant seeds used in this study were from a seedbank of the Department of Molecular Ecology, MPICE (http://www.ice.mpg.de/). The numbers of transgenic lines used in a glasshouse experiment are listed in Table S1. Plants grown in the field plot for the predation assay were the F12 generation of an advanced intercross-recombinant inbred line (AI-RIL) population [24], which was constructed from a cross between two inbred lines separately originating from collections in Arizona (AZ) and Utah (UT) by Baldwin and colleagues [79, 80]. The field experiments were performed at the Walnut Creek Center for Education and Research (WCCER)(34°55′17.8″N 112°50′42.2″W) located in Arizona, southwestern United States. The plants grown in the field are native to the area, not endangered species, non-transgenic material and non-regulated. No samples were collected from the wild. The field experiments were permitted and supported by WCCER. Seeds were germinated on Gamborg’s B5 medium in Petri dishes under conditions previously described by Krügel and colleagues [81]. Two weeks later, seedlings were transferred into peat plugs (Jiffy 703, www.jiffypot.com). During the next 2 weeks seedlings were increasingly exposed to outdoor conditions and subsequently transplanted into the field plot. The plants were watered regularly by a drip irrigation system [82].

Plants used for the different light treatments and dynamic volatile collections were grown in a glasshouse. The generation and screening of the transgenic lines (listed in Table S1) silenced in the expression of photoreceptor genes and *NaMPK4* were previously described [31, 34]. Seedlings were germinated in the described way and transferred into potting soil (www.klasmann-deilmann.com) in small pots (TEKU JJP 3050 104 pots, Poeppelmann GmbH & Co. KG, Lohne, Germany) for 10 days and then transplanted into 1 L pots and kept in a glasshouse (19 °C–35 °C, 55% humidity, light period: 6:00–22:00, supplemental lighting by Philips Sun-T Agro 400 W and 600 W sodium lights) [81, 83]. Plants were watered and fertilized as previously described by Schuman et al. [84]. Experimental research and field studies on plants complies with relevant institutional, national, and international guidelines and legislation.

### *M. sexta* rearing and regurgitant collection

*M. sexta* used in this study were from an in-house colony maintained on artificial diet (Bell RA & Joachim FG 1976) in a climate chamber (24 °C, light period: 0:00 to 13:00, relative humidity: 70%). For collection of regurgitant, caterpillars were reared on *N. attenuata* UT WT plants. Collection and storage of regurgitant were previously described [78, 85].

### W + R treatment and headspace sampling

The first stem leaf of elongated plants was chosen for elicitation with wounding by a pattern wheel and the immediate addition of 20 μL of 20% diluted regurgitant in water to the puncture wounds in the leaf lamina. The treated leaf was gently rubbed with fingers in clean gloves. This leaf was enclosed in a ventilated PET cup (ca. 650 mL). Headspace volatiles were collected as described by Kallenbach et al. [30] with two pieces of silicone laboratory tubing (ST, 1 mm i.d. × 1.8 mm o.d.; Carl Roth, catalog number: 9555.1; 5 mm for each piece) placed into the cup under the leaf for 4 h (time-resolved) or 24 h (long term collection). For the quantification of internal volatile pools, the sampled leaf was flash-frozen and ground in liquid N_2_. One hundred mg tissue was placed into a 1.5 mL glass GC vial (Sigma-Aldrich) and 0.8 mL saturated CaCl_2_ solution was added. One piece of ST was placed into the vial and fully immersed into the solution to extract free volatiles [29]. STs with collected volatiles were stored in a clean glass vial in − 20 °C freezer.

### Light deprivation, far-red supplementation and ABA treatment

Temporary, local light deprivation was applied to elicited leaves by wrapping the collection cup with aluminum foil. Temperature and humidity in the glasshouse, clean and wrapped cup were recorded by PRO-USB-2 data Logger with humidity, temperature sensor (RS Components GmbH). Additional far-red was supplied as previously described [31]: five-Watt far-red light emitting diodes (LED, 720 ± 10 nm) were directed to the adaxial side of the targeted leaf. ABA (Sigma-Aldrich) was diluted to 2 μM and sprayed on the leaf in the morning at around 6:00. Stomatal resistance was measured by an AP4 Porometer (AP4-UM-3, Delta-T Devices) following the manufacturer’s instructions.

### TD–GC–QMS analysis

STs with absorbed volatiles were analyzed on a quadrupole GC-MS-QP2010Ultra equipped with a TD-20 thermal desorption unit (Shimadzu). Desorption and analysis were performed as previously described [30]. Peaks were integrated using the target and reference ions as previously described [23] and tentative compound identifications were based on comparison with an in-house library of *N. attenuata* volatiles prepared in the same lab including the spectra, retention times and relative retention orders of these volatiles from standards and samples analyzed using the same method. Identification of volatiles in the reference library was based on pure standards, Kovats retention indices and comparison of spectra to NIST MS libraries [30].

### Measuring transcript abundance of genes

The transcription levels of *NaLIS, NaGPPS1* and *NaGPPS2* in leaves under standard light or light deprivation conditions were quantified using qPCR as previously described [23]. Total RNA was extracted using the NucleoSpin® RNA Plant kit (MACHEREY-NAGEL). After digestion of genomic DNA, 1 μg total RNA was transcribed with the PrimeScript™ RT reagent Kit (TAKARA). Primer sequences were described previously [23] and the qPCR was run in a MX3005P PCR cycler (Stratagene).

### Identification of *cis*-acting regulatory elements

The 2Kb sequences upstream of the start codons of *NaLIS*, *NaTPS38*, *NaHPL* and *NaLOX2* were extracted from the genomic sequences of *N. attenuata* [86]. Then these sequences were searched online through the Plant *Cis*-acting Regulatory Element database (http://bioinformatics.psb.ugent.be/webtools/plantcare/html/, Lescot et al. [2002]).

## Supplementary Information

**Additional file 1 Fig. S1.** Diurnal predation of eggs and larvae of *M. sexta* feeding on *N. attenuata* plants. (A) Diagram of natural predators (like *Geocoris* spp.) capture preys (such as *M. sexta* larvae and eggs) on *N. attenuata* plants. (B) Number of predated eggs and larvae of *M. sexta* observed in a field plot of *N. attenuata* plants during day or night periods. **Fig. S2.** Difference in temperature and humidity caused by wrapping the collecting cups. **Fig. S3.** Volatile emissions in plants with *NaCrypt* and *NaCrypt2* silenced (related to Fig. 2). **Fig. S4.** Supplementation with extra far-red light did not alter the dynamics of volatile emissions. **Table S1.** Transgenic lines used in this study. **Table S2.** Detailed information of *cis*-acting regulatory elements identified in the promoter sequences of *NaLIS*, *NaTPS38*, *NaHPL* and *NaLOX.*

## Data Availability

The datasets used and/or analysed during the current study are available from the corresponding author on reasonable request.

## Abbreviations

ABA: Abscisic acid
Crypt: Cryptochrome
GLV: Green leaf volatiles
GPPS: Geranylgeranyl diphosphate synthase
HIPV: Herbivore-induced plant volatile
HPL: Hydroperoxide lyase
JA: Jasmonate
LIS: Linalool synthase
LOX: Lipoxygenase
MeJA: Methyl jasmonate
MPK4: Mitogen-activated protein kinase 4
PhyA: Phytochrome A
PhyB: Phytochrome B
SA: Salicylic acid
TPS: Terpene synthase
W + R: Wounding plus *M. sexta* larval regurgitant
