# Supplementary material for: Light dominates the diurnal emissions of herbivore-induced volatiles in wild tobacco
Source: BMC Plant Biol. 2021 Aug 30;21:401. doi: 10.1186/s12870-021-03179-z (PMC8404343; doi:10.1186/s12870-021-03179-z)
Supplement: Supplementary file 1 — Additional file 1 Fig. S1. Diurnal predation of eggs and larvae of M. sexta feeding on N. attenuata plants. (A) Diagram of natural predators (like Geocoris spp.) capture preys (such as M. sexta larvae and eggs) on N. attenuata plants. (B) Number of predated eggs and larvae of M. sexta observed in a field plot of N. attenuata plants during day or night periods. Fig. S2. Difference in temperature and humidity caused by wrapping the collecting cups. Fig. S3. Volatile emissions in plants with NaCrypt and NaCrypt2 silenced (related to Fig. 2). Fig. S4. Supplementation with extra far-red light did not alter the dynamics of volatile emissions. Table S1. Transgenic lines used in this study. Table S2. Detailed information of cis-acting regulatory elements identified in the promoter sequences of NaLIS, NaTPS38, NaHPL and NaLOX. [file 12870_2021_3179_MOESM1_ESM.docx]

**Supplementary material**

**Title:**

**Light dominates the diurnal emissions of herbivore-induced volatiles in wild tobacco**

**Authors**: Jun He^a,b*^, Rayko Halitschke^b^, Meredith C. Schuman^b,c^, Ian T. Baldwin^b*^

^a^Current address: National Citrus Engineering Research Center, Citrus Research Institute, Southwest University, Chongqing 400712, P. R. China

^b^Department of Molecular Ecology, Max Planck Institute for Chemical Ecology, 07745, Jena, Germany

^c^Current address: departments of Geography and Chemistry, University of Zurich, 8057 Zürich, Switzerland

*To whom correspondence should be addressed

**Figure S1**. **Diurnal predation of eggs and larvae of *M. sexta* feeding on *N. attenuata* plants.** (**A**) Schematic of natural predators (such as *Geocoris* spp.) that attack *Manduca sexta* larvae and eggs on *N. attenuata* plants. (**B**) Number of predated eggs and larvae of *M. sexta* observed in a field plot of *N. attenuata* plants during day or night periods.


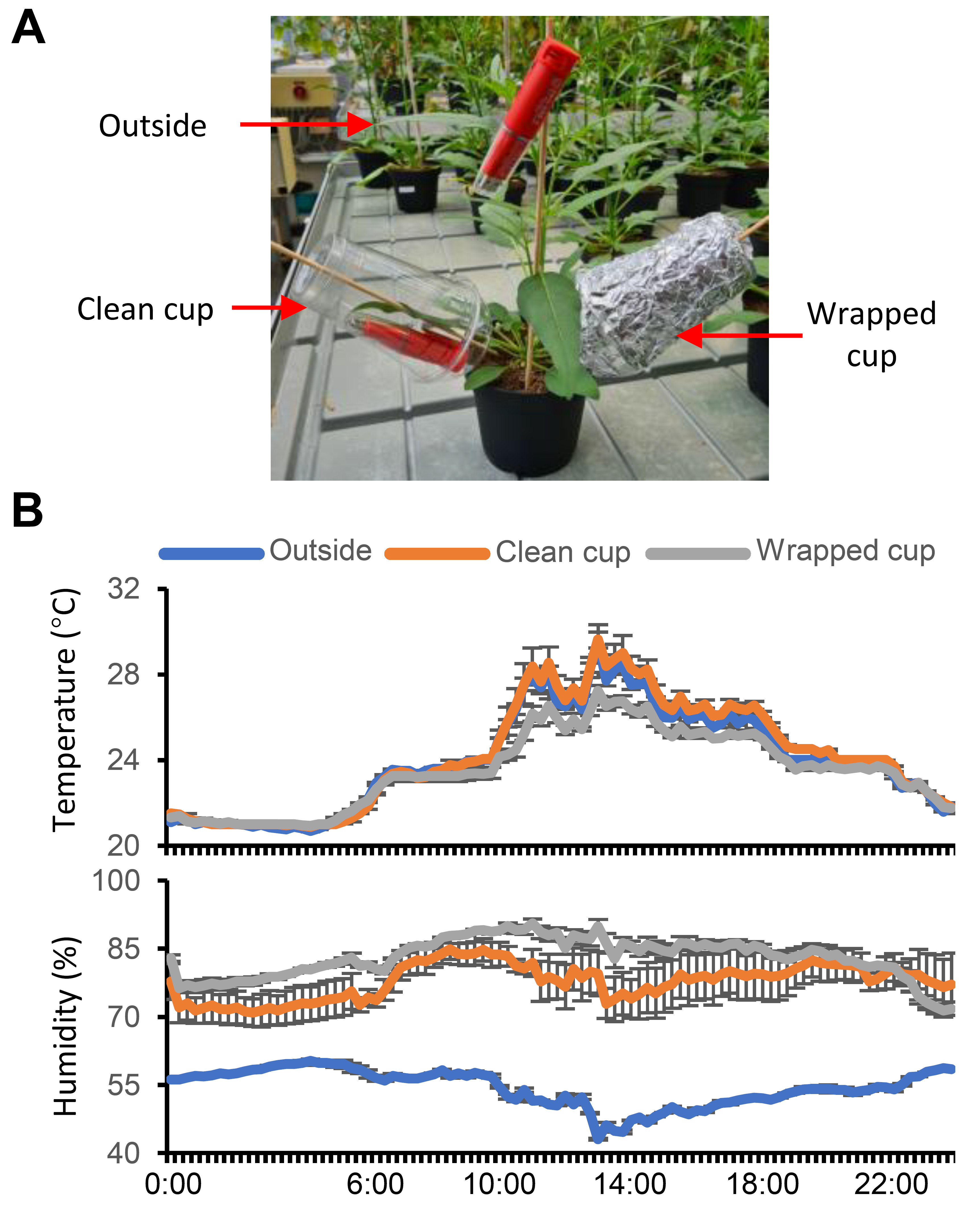


**Figure S2. Difference in temperature and humidity caused by wrapping the collecting cups.** (**A**) Setup of the measurements. Three data loggers with temperature and humidity sensors were placed outside, in a clean cup and in a wrapped cup to record temperature and humidity every 15 minutes for 24 hours. The experiment was repeated twice. (**B**) The temporary temperature and humidity in the glasshouse but outside of the cups, in clean cups and in wrapped cups.


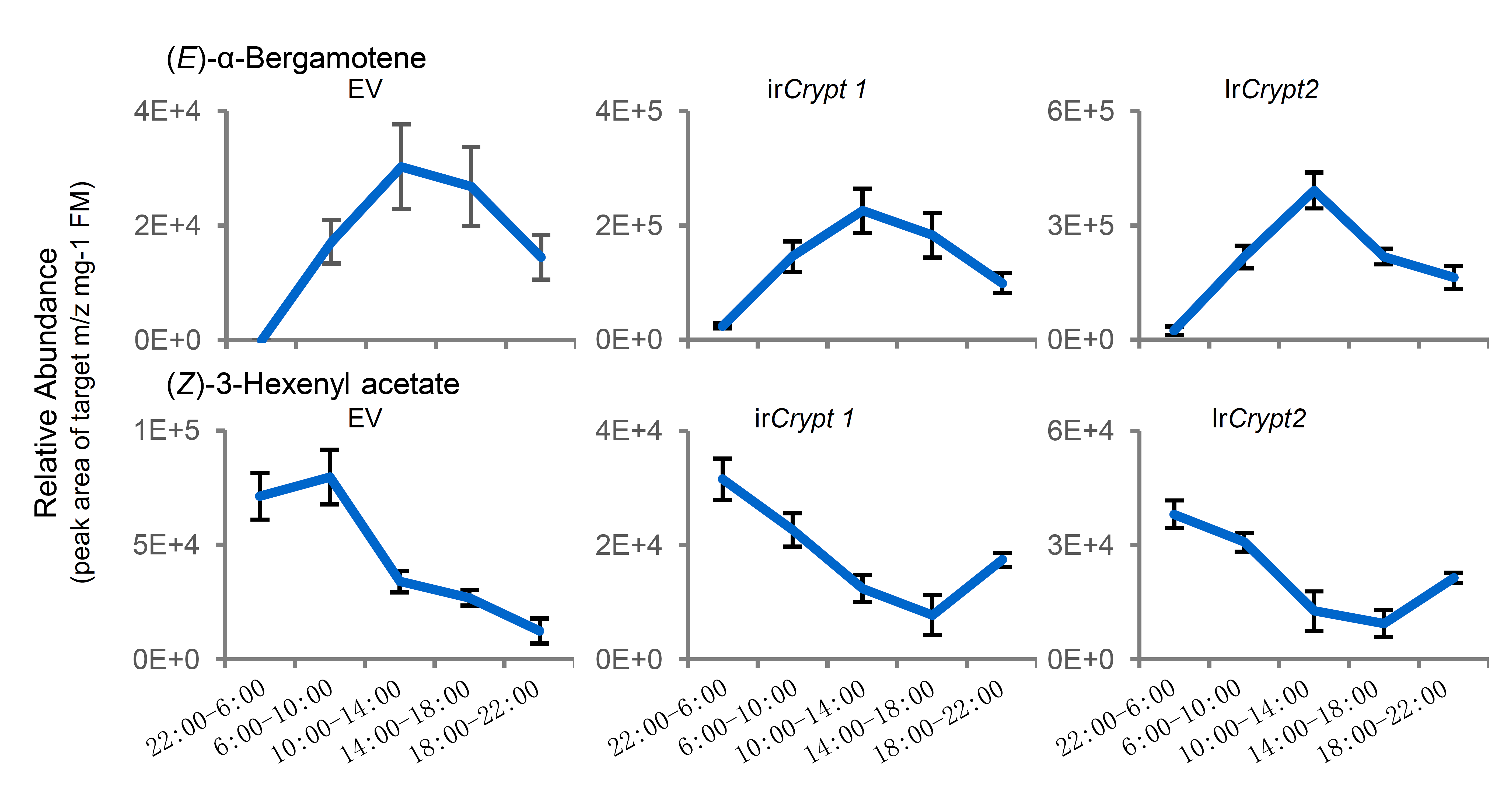


**Figure S3. Volatile emissions in plants with *NaCrypt* and *NaCrypt2* silenced by RNAi.**


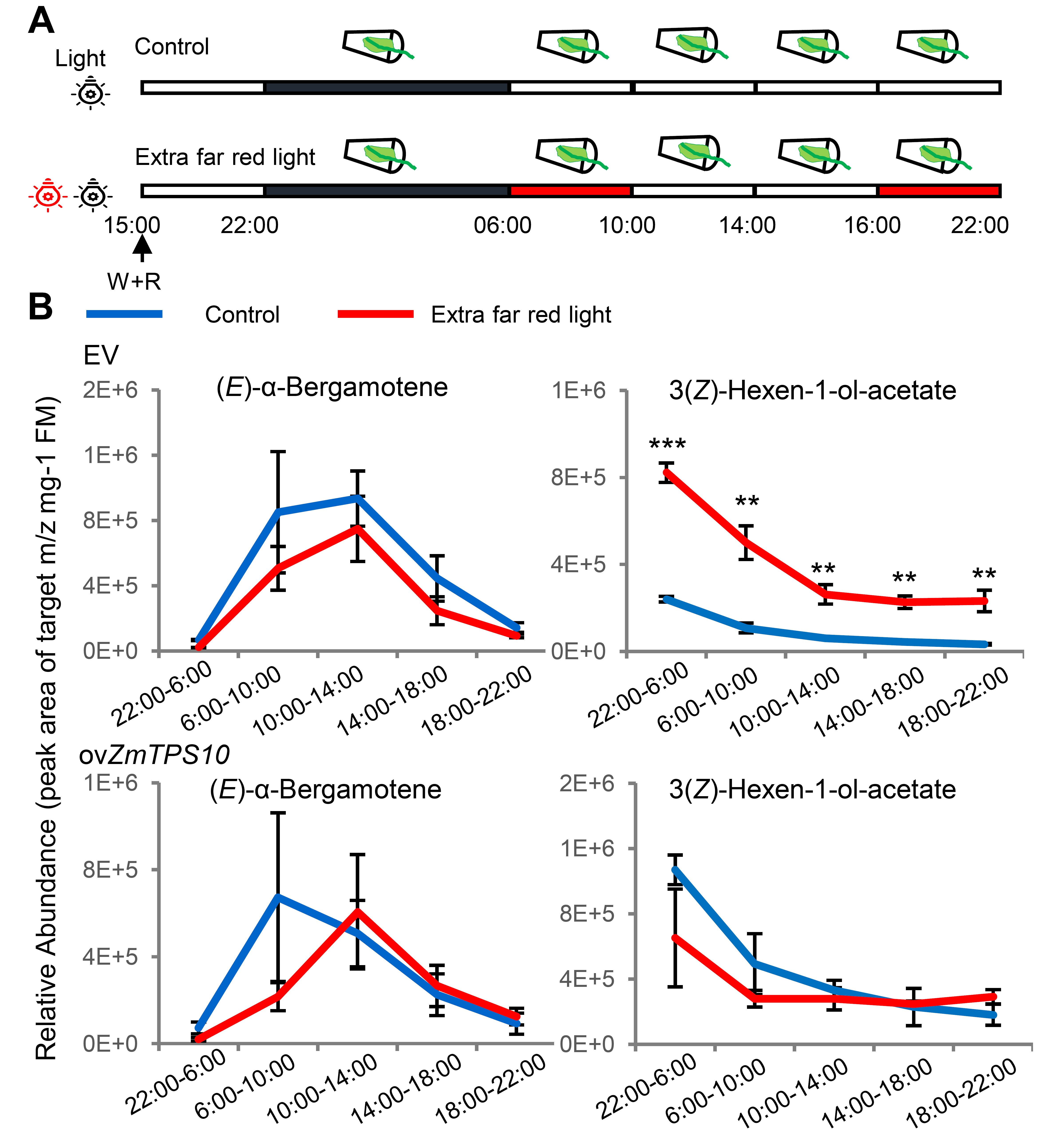
**Figure S4. Supplementation with extra far-red light did not alter the dynamics of volatile emissions.** (**A**) Experimental set-up for far red light treatments and volatile collections after W+R treatment during night and day. (**B**) Dynamic induction of representative volatiles by W+R treatment and supplementation of extra far red light. (Student *t*-test, n=3, **: p<0.05, ***: p<0.001).

| **Table S1.** Transgenic lines used in this study. | | |
| --- | --- | --- |
| Abbreviation | Line number | Target of gene* |
| ir*PhyB1* | A-12-178-1 | *NaPhyA* |
| ir*PhyB2* | A-12-204-2 | *NaPhyA* |
| ir*PhyB1xirPhyB2* | A-12-178-1 x A-12-204-2 | *NaPhyB1,NaPhyB2* |
| ir*Cryp1* | A-12-303-4-8 | *NaCryp1* |
| ir*Cryp2* | A-12-282-3-9 | *NaCryp2* |
| ir*MPK4* | A-08-119 | *NaMPK4* |

*, ir*MPK4* line was characterized by Hettenhausen et al. (2012). All other lines were characterized by Oh et al. (2018).

| **Table S2.** Detailed information of *cis*-acting regulatory elements identified in the promoter sequences of *NaLIS, NaTPS38, NaHPL and NaLOX* | | | | | | |
| --- | --- | --- | --- | --- | --- | --- |
| Gene | Element | Sequence | Position | Matrix score | Strand | Function description |
| *NaLIS* | Box 4 | ATTAAT | 124 | 6 | + | part of a conserved DNA module involved in light responsiveness |
|  | MYB | CAACAG | 355 | 6 | + |  |
|  | Myc | TCTCTTA | 501 | 7 | + |  |
|  | MYC | CATGTG | 578 | 6 | + |  |
|  | Box 4 | ATTAAT | 764 | 6 | + | part of a conserved DNA module involved in light responsiveness |
|  | Box 4 | ATTAAT | 922 | 6 | + | part of a conserved DNA module involved in light responsiveness |
|  | AE-box | AGAAACAA | 993 | 8 | - | part of a module for light response |
|  | TGACG-motif | TGACG | 1080 | 5 | + | *cis*-acting regulatory element involved in the MeJA-responsiveness |
|  | circadian | CAAAGATATC | 1234 | 9 | - | *cis*-acting regulatory element involved in circadian control |
|  | ABRE | ACGTG | 1460 | 5 | - | *cis*-acting element involved in the abscisic acid responsiveness |
|  | G-box | CACGTC | 1460 | 6 | + | *cis*-acting regulatory element involved in light responsiveness |
|  | Myb | TAACTG | 1627 | 6 | + |  |
|  | MYC | CAATTG | 1732 | 6 | - |  |
|  | Box 4 | ATTAAT | 1828 | 6 | - | part of a conserved DNA module involved in light responsiveness |
|  |  |  |  |  |  |  |
| *NaTPS38* | AE-box | AGAAACAA | 14 | 8 | - | part of a module for light response |
|  | MYC | CAATTG | 391 | 6 | + |  |
|  | circadian | CAAAGATATC | 451 | 9 | + | *cis*-acting regulatory element involved in circadian control |
|  | chs-CMA1a | TTACTTAA | 690 | 8 | - | part of a light responsive element |
|  | MYC | CATTTG | 1016 | 6 | + |  |
|  | CGTCA-motif | CGTCA | 1495 | 5 | + | *cis*-acting regulatory element involved in the MeJA-responsiveness |
|  | TGACG-motif | TGACG | 1495 | 5 | - | *cis*-acting regulatory element involved in the MeJA-responsiveness |
|  | TCA-element | TCAGAAGAGG | 1594 | 9 | + | *cis*-acting element involved in salicylic acid responsiveness |
|  | Myb | CAACTG | 1639 | 6 | - |  |
|  | GT1-motif | GGTTAA | 1784 | 6 | - | light responsive element |
|  | MYB-like sequence | TAACCA | 1785 | 6 | + |  |
|  | TCT-motif | TCTTAC | 1802 | 6 | + | part of a light responsive element |
|  | Box 4 | ATTAAT | 1847 | 6 | - | part of a conserved DNA module involved in light responsiveness |
|  | MYC | CATGTG | 1937 | 6 | + |  |
|  |  |  |  |  |  |  |
| *NaHPL* | GA-motif | ATAGATAA | 118 | 8 | + | part of a light responsive element |
|  | I-box | AGATAAGG | 120 | 8 | + | part of a light responsive element |
|  | CGTCA-motif | CGTCA | 230 | 5 | + | *cis*-acting regulatory element involved in the MeJA-responsiveness |
|  | GT1-motif | GGTTAA | 266 | 6 | + | light responsive element |
|  | CGTCA-motif | CGTCA | 484 | 5 | + | *cis*-acting regulatory element involved in the MeJA-responsiveness |
|  | Box 4 | ATTAAT | 538 | 6 | + | part of a conserved DNA module involved in light responsiveness |
|  | MYC | CATTTG | 625 | 6 | + |  |
|  | Myc | TCTCTTA | 647 | 7 | - |  |
|  | MYC | CAATTG | 829 | 6 | + |  |
|  | ABRE | CACGTG | 849 | 6 | + | *cis*-acting regulatory element involved in light responsiveness |
|  | G-Box | CACGTG | 849 | 6 | + | *cis*-acting element involved in the abscisic acid responsiveness |
|  | G-box | CACGTG | 849 | 6 | + | *cis*-acting regulatory element involved in light responsiveness |
|  | Box 4 | ATTAAT | 952 | 6 | + | part of a conserved DNA module involved in light responsiveness |
|  | MBS | CAACTG | 1148 | 6 | - |  |
|  | Myb | CAACTG | 1148 | 6 | - | MYB binding site involved in drought-inducibility |
|  | CGTCA-motif | CGTCA | 1152 | 5 | - | *cis*-acting regulatory element involved in the MeJA-responsiveness |
|  | Box 4 | ATTAAT | 1174 | 6 | - | part of a conserved DNA module involved in light responsiveness |
|  | MYC | CATTTG | 1504 | 6 | - |  |
|  | ABRE | AACCCGG | 1535 | 7 | - | *cis*-acting element involved in the abscisic acid responsiveness |
|  | G-box | CACGAC | 1681 | 6 | + | *cis*-acting regulatory element involved in light responsiveness |
|  | MYC | CATGTG | 1993 | 6 | - | part of a light responsive element |
|  |  |  |  |  |  |  |
| *NaLOX* | TC-rich repeats | GTTTTCTTAC | 235 | 9 | - | *cis*-acting element involved in defense and stress responsiveness |
|  | TCT-motif | TCTTAC | 354 | 6 | - | part of a light responsive element |
|  | MBS | CAACTG | 440 | 6 | + | MYB binding site involved in drought-inducibility |
|  | CGTCA-motif | CGTCA | 473 | 5 | + | *cis*-acting regulatory element involved in the MeJA-responsiveness |
|  | MYC | CATGTG | 564 | 6 | + |  |
|  | MYC | CATGTG | 571 | 6 | - |  |
|  | CGTCA-motif | CGTCA | 602 | 5 | + | *cis*-acting regulatory element involved in the MeJA-responsiveness |
|  | MYC | CAATTG | 685 | 6 | + |  |
|  | ABRE | CACGTG | 743 | 6 | + | *cis*-acting element involved in the abscisic acid responsiveness |
|  | G-Box | CACGTG | 743 | 6 | + | *cis*-acting regulatory element involved in light responsiveness |
|  | G-box | CACGTG | 743 | 6 | + | *cis*-acting regulatory element involved in light responsiveness |
|  | ABRE | ACGTG | 744 | 5 | + | *cis*-acting element involved in the abscisic acid responsiveness |
|  | ABRE | CACGTG | 778 | 6 | + | *cis*-acting element involved in the abscisic acid responsiveness |
|  | G-Box | CACGTG | 778 | 6 | + | *cis*-acting regulatory element involved in light responsiveness |
|  | G-box | CACGTG | 778 | 6 | + | *cis*-acting regulatory element involved in light responsiveness |
|  | ABRE | ACGTG | 779 | 5 | + | *cis*-acting element involved in the abscisic acid responsiveness |
|  | Box 4 | ATTAAT | 961 | 6 | + | part of a conserved DNA module involved in light responsiveness |
|  | MYC | CATGTG | 968 | 6 | - |  |
|  | Box 4 | ATTAAT | 1032 | 6 | - | part of a conserved DNA module involved in light responsiveness |
|  | Box 4 | ATTAAT | 1041 | 6 | - | part of a conserved DNA module involved in light responsiveness |
|  | ABRE | ACGTG | 1132 | 5 | - | *cis*-acting element involved in the abscisic acid responsiveness |
|  | G-Box | CACGTT | 1132 | 6 | + | *cis*-acting regulatory element involved in light responsiveness |
|  | MYB | TAACCA | 1289 | 6 | - |  |
|  | GT1-motif | GGTTAA | 1290 | 6 | + | light responsive element |
|  | ACE | GACACGTATG | 1301 | 9 | + | *cis*-acting element involved in light responsiveness |
|  | Box 4 | ATTAAT | 1358 | 6 | - | part of a conserved DNA module involved in light responsiveness |
|  | Box 4 | ATTAAT | 1402 | 6 | - | part of a conserved DNA module involved in light responsiveness |
|  | Box 4 | ATTAAT | 1603 | 6 | - | part of a conserved DNA module involved in light responsiveness |
|  | ABRE | ACGTG | 1773 | 5 | - | *cis*-acting element involved in the abscisic acid responsiveness |
|  | G-Box | CACGTT | 1773 | 6 | + | *cis*-acting regulatory element involved in light responsiveness |
|  | G-box | TAAACGTG | 1773 | 8 | - | *cis*-acting regulatory element involved in light responsiveness |
|  | circadian | CAAAGATATC | 1835 | 9 | + | *cis*-acting regulatory element involved in circadian control |
|  | GT1-motif | GGTTAA | 1950 | 6 | - | light responsive element |
